# Antidepressant-Like Activity of Typical Antidepressant Drugs in the Forced Swim Test and Tail Suspension Test in Mice Is Augmented by DMPX, an Adenosine A_2A_ Receptor Antagonist

**DOI:** 10.1007/s12640-018-9959-2

**Published:** 2018-09-28

**Authors:** Ewa Poleszak, Aleksandra Szopa, Karolina Bogatko, Elżbieta Wyska, Sylwia Wośko, Katarzyna Świąder, Urszula Doboszewska, Aleksandra Wlaź, Andrzej Wróbel, Piotr Wlaź, Anna Serefko

**Affiliations:** 1 Department of Applied Pharmacy, Medical University of Lublin, Chodźki 1, PL 20-093 Lublin, Poland; 2 Department of Pharmacokinetics and Physical Pharmacy, Collegium Medicum, Jagiellonian University, Medyczna 9, PL 30-688 Kraków, Poland; 3 Department of Animal Physiology, Institute of Biology and Biochemistry, Faculty of Biology and Biotechnology, Maria Curie-Skłodowska University, Akademicka 19, PL 20-033 Lublin, Poland; 4 Department of Pathophysiology, Medical University of Lublin, Jaczewskiego 8, PL 20-090 Lublin, Poland; 5 Second Department of Gynecology, Medical University of Lublin, Jaczewskiego 8, PL 20-090 Lublin, Poland

**Keywords:** DMPX, Antidepressants, Forced swim test, Tail suspension test, Mice

## Abstract

Unsatisfactory therapeutic effects of currently used antidepressants force to search for new pharmacological treatment strategies. Recent research points to the relationship between depressive disorders and the adenosinergic system. Therefore, the main goal of our studies was to evaluate the effects of DMPX (3 mg/kg, i.p.), which possesses selectivity for adenosine A_2A_ receptors versus A_1_ receptors, on the activity of imipramine (15 mg/kg, i.p.), escitalopram (2.5 mg/kg, i.p.), and reboxetine (2 mg/kg, i.p.) given in subtherapeutic doses. The studies carried out using the forced swim and tail suspension tests in mice showed that DMPX at a dose of 6 and 12 mg/kg exerts antidepressant-like effect and does not affect the locomotor activity. Co-administration of DMPX at a dose of 3 mg/kg with the studied antidepressant drugs caused the reduction of immobility time in both behavioral tests. The observed effect was not associated with an increase in the locomotor activity. To evaluate whether the observed effects were due to a pharmacokinetic/pharmacodynamic interaction, the levels of the antidepressants in blood and brain were measured using high-performance liquid chromatography. It can be assumed that the interaction between DMPX and imipramine was exclusively pharmacodynamic in nature, whereas an increased antidepressant activity of escitalopram and reboxetine was at least partly related to its pharmacokinetic interaction with DMPX.

## Introduction

Adenosine is widely distributed throughout the central nervous system (CNS). It functions as an endogenous agonist active at purinoreceptors (Berk et al. 2001). Adenosine modulates neuronal function via controlling the release of various neurotransmitters (Sebastião and Ribeiro 2009), e.g., it highly inhibits dopamine (DA), γ-aminobutyric acid (GABA), glutamate (Glu), acetylcholine (ACh), serotonin (5-HT), and noradrenaline (NA) release (Sebastião and Ribeiro 1996). The adenosine receptors (ARs) belong to the G protein coupled receptor superfamily (Fredholm et al. 2000; Olah and Stiles 2000). At least four ARs subtypes are known: A_1_, A_2A_, A_2B_, and A_3_, which are d istributed in different organ systems and control various physiological functions in the human body (Olah and Stiles 1992, 2000; Fredholm et al. 2000, 2001). A_1_R and A_2A_R are mainly located in the CNS (especially in the cortex and striatum, respectively), whereas both A_2B_R and A_3_R are present in the brain in low amount (Corset et al. 2000).

Studies aimed at unraveling the neurobiological basis of depression and the mechanisms underlying antidepressant effects of drugs emphasize the role of the adenosinergic system in the regulation of mood (Cunha et al. 2008; Lara 2010; Gomes et al. 2011). Because the adenosinergic system modulates neurotransmission its impact on depressive behavior is complex (Scaccianoce et al. 1989; Chau et al. 1999; Okada et al. 2001; Yamato et al. 2002). Depressant-like effect of adenosine and its analogues has been demonstrated in animal behavioral despair tests (Minor et al. 1994; Hunter et al. 2003). Moreover, the administration of classic antidepressants counteracted this effect (Kulkarni and Mehta 1985). The results of preclinical investigations highlight in particular the relationship between the manipulation of A_2A_R and depression and suggest that A_2A_R antagonists may constitute a novel strategy for the treatment of depressive disorders (Cunha 2008; Gomes et al. 2011). El Yacoubi et al. (2001, 2003) demonstrated that A_2A_R knockout mice are predisposed to the occurrence of antidepressant-like behavior. Likewise, Cunha et al. (2006) observed that blockade of these receptors inhibits changes in the hippocampus caused by one of the major environmental factors conducive to depression, i.e., stress. Furthermore, the therapeutic strategies currently used in depressed patients also affect the adenosinergic system (e.g., desipramine, chlorimipramine, nortriptiline, tricyclic antidepressant drugs (TCAs), are able to bind to the ARs) (Deckert and Gleiter 1989).

Because of an increasing evidence that adenosine neurotransmission is engaged in the development of psychiatric disorders, including major depressive disorder (Kaster et al. 2015; Ortiz et al. 2015; Ali-Sisto et al. 2016), and in the mechanisms underlying antidepressant effects (Deckert and Gleiter 1989; Fredholm et al. 1999; Cunha et al. 2008; Lara 2010; Gomes et al. 2011), while the number of prescribed drugs is growing (Murray and Lopez 1997; Tondo et al. 2003; Hashimoto 2011), the possible interaction between selective A_2A_R antagonist and commonly used antidepressants should be examined. Therefore, we assessed the antidepressant-like effect of DMPX, a synthetic analog of caffeine, which possesses higher selectivity for A_2A_ receptors versus A_1_ receptors (Armentero et al. 2011), in two widely used preclinical screening tests, the forced swim test (FST) (Porsolt et al. 1977) and tail suspension test (TST) (Steru et al. 1985). In the next step, we examined the effects of DMPX on the antidepressant activity of a TCA, a selective 5-HT reuptake inhibitor (SSRI), and a selective NA reuptake inhibitor (SNRI), imipramine, escitalopram, and reboxetine, respectively. To exclude false positive outcomes obtained in the FST and TST, animal’s spontaneous locomotor activity was measured. Further, to evaluate whether the observed effects during short-term exposure to inescapable and uncontrollable stress were due to a pharmacokinetic/pharmacodynamic interaction, the levels of the studied antidepressants in the collected biological material (blood and brain tissue) were measured using a high-performance liquid chromatography (HPLC) method.

## Materials and Methods

### Animals

The experiment was carried out on 336 naïve adult male albino Swiss mice weighing 25–30 g, purchased from the licensed breeder (Kołacz, Warsaw, Poland). The animals were housed in the environmentally controlled rooms (temperature maintained at 21–25 °C and humidity 40–60%) in standard cages in groups of 10 with unlimited access to water and food. The rooms were illuminated with a 12-h light/dark cycle. The procedures began after at least 1-week acclimation period in the laboratory conditions and were performed between 8 a.m. and 3 p.m. to minimize circadian influences. Behavioral tests were video recorded and then analyzed by two blind experimenters. All procedures were conducted in accordance with the European Communities Council Directive and Polish legislation acts concerning animal experimentations. The procedures and protocols were approved by the First Local Ethics Committee at the Medical University of Lublin (license no. 5/2015).

### Drug Administration

DMPX (3,7-dimethyl-1-propargylxanthine, 3, 6, and 12 mg/kg, Sigma-Aldrich, Poznań, Poland) was suspended in a 1% aqueous solution of Tween 80 (POCH S.A., Gliwice, Poland). Imipramine hydrochloride (15 mg/kg, Sigma-Aldrich), reboxetine mesylate (2.5 mg/kg, Ascent Scientific, Cambridge, UK), and escitalopram oxylate (2 mg/kg, Sigma-Aldrich) were dissolved in 0.9% NaCl. All solutions of antidepressants were administered intraperitoneally (i.p.) 60 min, whereas DMPX suspension was injected i.p. 30 min prior behavioral testing. The volume of all administered solutions/suspension was 0.01 ml/g. The doses and treatment schedules were selected on the basis of literature and the results of our previous experiments (Poleszak 2007; Poleszak et al. 2005, 2011, 2013, 2016a,b; Szewczyk et al. 2002, 2009; Szopa et al. 2016). Animals from the control groups received i.p. injections of saline.

### FST

FST was carried out according to the method of Porsolt et al. (1977). Each mouse was placed individually for 6 min into a glass cylinder (height 25 cm, diameter 10 cm) with 15 cm of water at 23–25 °C. After the first 2 min of the test, total duration of immobility (in seconds) was measured. An animal was judged to be immobile when it ceased struggling and remained floating motionless and making only movements allowing to keep the head just above the surface of water.

### TST

TST was carried out according to the method of Steru et al. (1985). Each mouse was suspended for 6 min by the tail (2 cm from the end of the tail) using adhesive tape. After the first 2 min of the test, total duration of immobility (in seconds) was measured. An animal was judged to be immobile when it ceased moving limbs and body, making only movements allowing to breathe.

### Spontaneous Locomotor Activity

Spontaneous locomotor activity was assessed using Opto-Varimex-4 Auto-Track (Columbus Instruments, Columbus, OH, USA). Plexiglas cages with lids (43 × 43 × 32 cm) were equipped with a set of four infrared emitters and four detectors monitoring mice movements. Each animal was placed individually for 6 min into a cage to measure the distance (in cm) traveled between the second and the sixth minutes, which corresponds to the time interval analyzed in the FST and TST.

### Determination of Antidepressant Levels in Serum and Brain Homogenates

To acquire blood and brain for pharmacokinetic studies, mice were decapitated in an appropriate time after injection of examined drugs with or without DMPX. The blood was collected into Eppendorf tubes and allowed to cloth. Then the samples were centrifuged for 10 min at 1000 rpm and the serum was collected into polyethylene tubes and frozen at − 25 °C. Brains, just after decapitation, were dissected from the skulls, rinsed with 0.9% NaCl, and frozen at − 25 °C.

Brain and serum concentrations of antidepressants were assayed by HPLC method as it was described previously (Poleszak et al. 2016a; Szopa et al. 2016).

Calibration curves that were developed on the basis of the ratio of the peak heights of the tested compounds to internal standard versus the concentration of the drug were linear in the tested concentration ranges. No interfering peaks were observed in the chromatograms. The assays were reproducible with low intra- and interday variation (a coefficient of variation less than 10%). The extraction efficiencies of the analyzed compounds and internal standards ranged from 66 to 97%. Concentrations of antidepressants were expressed in ng/ml of serum and ng/g of wet brain tissue.

### Statistical Analysis

Statistical analysis was performed using one-way analysis of variance (ANOVA) with Dunnett’s post hoc test, two-way ANOVA with Bonferroni’s post hoc test, or Student’s *t* test, depending on the study design. All results are presented as mean  ± SEM for each experimental group. *P* values < 0.05 were considered statistically significant.

## Results

### FST

#### DMPX Dose-Effect Relationship in FST

**Fig. 1:**
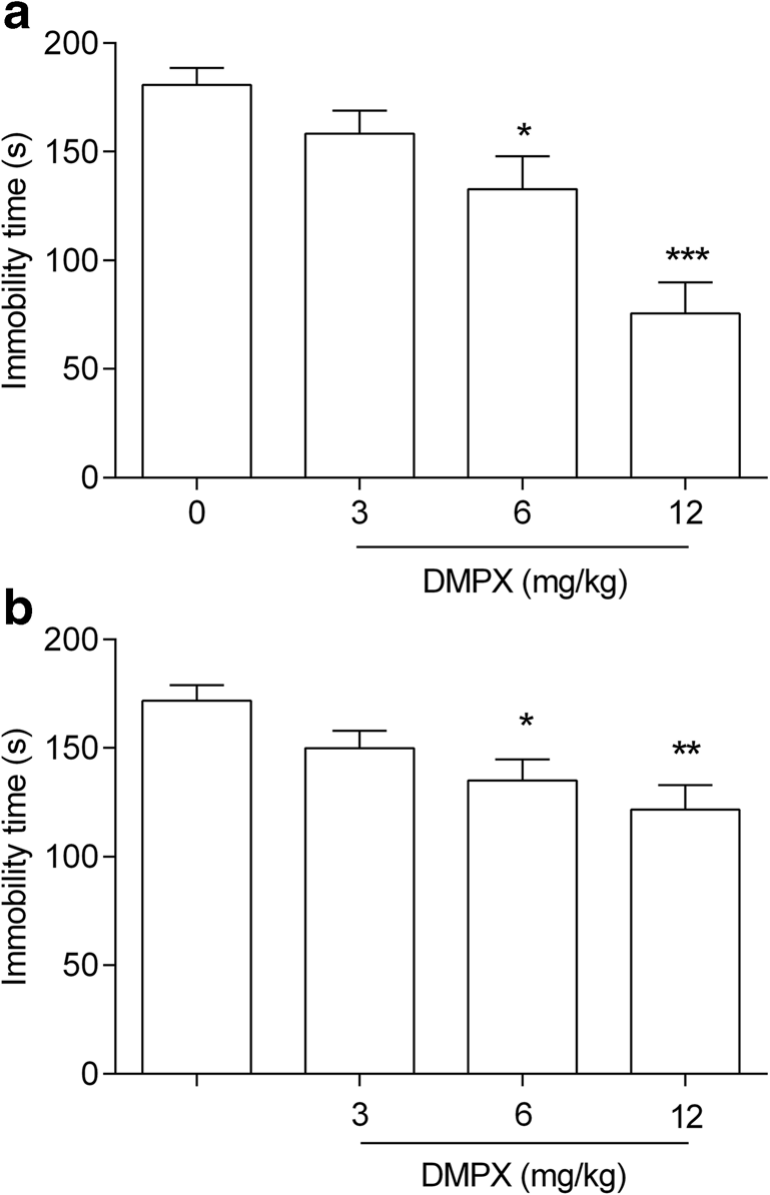
The antidepressant-like activity of DMPX in the FST (**a**) and TST (**b**) in mice. DMPX and saline were administered i.p. 30 min before the test. Each experimental group consisted of 10 animals. The data are presented as the means ± SEM. *p < 0.05, **p < 0.01, ***p < 0.001 versus control group (one-way ANOVA followed by Dunnett’s post hoc test)

In dose-effect studies, DMPX was used in three different doses: 3, 6, and 12 mg/kg (Fig. 1a). The statistical analysis of the FST results showed that DMPX at doses of 6 and 12 mg/kg exhibited an antidepressant-like activity. DMPX at a dose of 3 mg/kg had no statistically significant influence on mice behavior in the FST (one-way ANOVA: *F*(3,36) = 13.48, *p < 0.05, ***p < 0.001, *p* > 0.05, respectively).

#### Effect of Combined Administration of DMPX and Tested Antidepressants in FST

##### Effect of Combined Administration of DMPX and Imipramine in FST

**Fig. 2:**
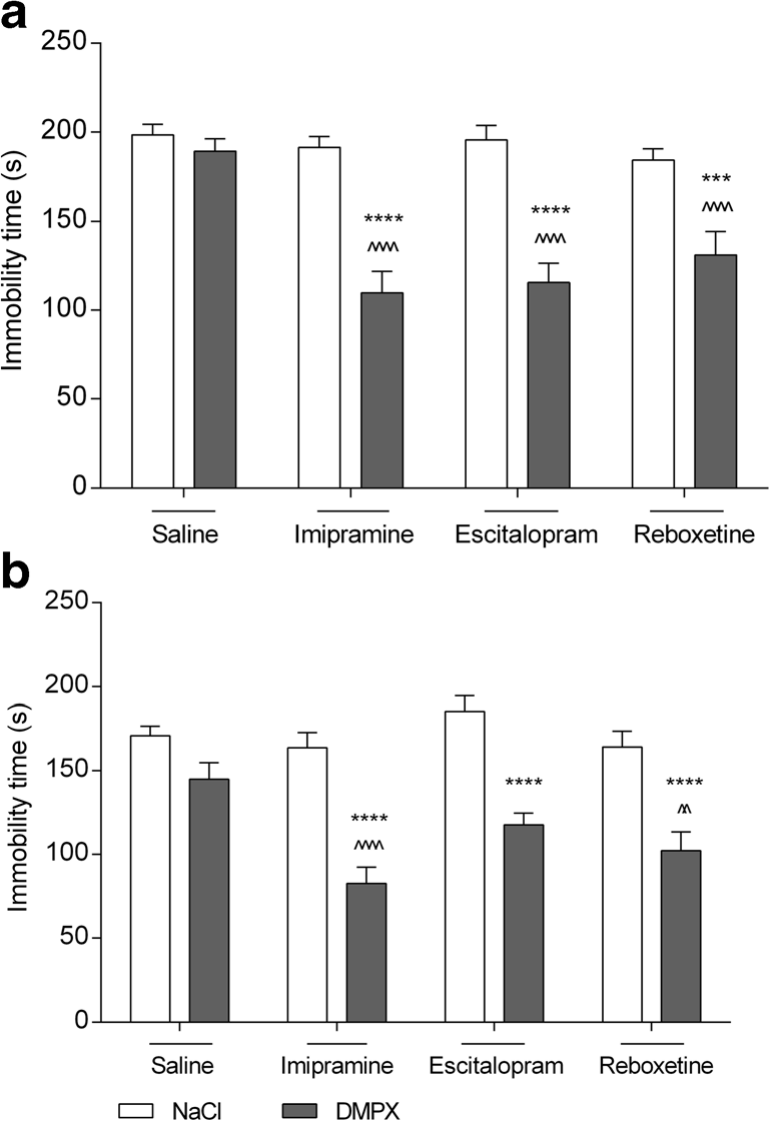
Effect of combined administration of DMPX and antidepressants in the FST (**a**) and TST (**b**) in mice. Antidepressants and saline were administered i.p. 60 min, whereas DMPX i.p. 30 min before the test. Each experimental group consisted of 10 animals. The data are presented as the means ± SEM. ***p < 0.001, ****p < 0.0001 versus respective antidepressant-treated group; ^^*p* < 0.01, ^^^^*p* < 0.0001 versus DMPX-treated group (two-way ANOVA followed by Bonferroni’s post hoc test)

DMPX (3 mg/kg) and imipramine (15 mg/kg), administered alone, did not affect the immobility in the FST (*p* > 0.05) in mice. However, when given together, the duration of mouse immobility was significantly shortened (*p* < 0.0001) (Fig. 2a).

Two-way ANOVA showed a significant effect of imipramine (*F*(1,36) = 29.18, *p* < 0.001), a significant effect of DMPX (*F*(1,36) = 32.23, *p* < 0.001), and a significant interaction between imipramine and DMPX (*F*(1,36) = 20.83, *p* < 0.001).

##### Effect of Combined Administration of DMPX and Escitalopram in FST

DMPX (3 mg/kg) and escitalopram (2 mg/kg), administered alone, did not affect the immobility in the FST (*p* > 0.05) in mice. However, when given together, the duration of mouse immobility was significantly shortened (*p* < 0.001) (Fig. 2a).

Two-way ANOVA showed a significant effect of escitalopram (*F*(1,35) = 23.20, *p* < 0.001), a significant effect of DMPX (*F*(1,35) = 31.16, *p* < 0.001), and no interaction between escitalopram and DMPX (*F*(1,35) = 19.94, *p* < 0.001).

##### Effect of Combined Administration of DMPX and Reboxetine in FST

DMPX (3 mg/kg) and reboxetine (2.5 mg/kg), administered alone, did not affect the immobility in the FST (*p* > 0.05) in mice. However, when given together, the duration of mouse immobility was significantly shortened (*p* < 0.0001) (Fig. 2a).

Two-way ANOVA demonstrated a significant effect of reboxetine (*F*(1,36) = 17.88, *p* = 0.0002), a significant effect of DMPX (*F*(1,36) = 13.27, *p* = 0.0008), and a significant interaction between reboxetine and DMPX (*F*(1,36) = 6.772, *p* = 0.0134).

### TST

#### DMPX Dose-Effect Relationship in TST

In dose-effect studies, DMPX was used in three different doses: 3, 6, and 12 mg/kg (Fig. 1b). The statistical analysis of the TST results showed that DMPX at a dose of 6 and 12 mg/kg had an antidepressant-like activity, which was manifested by the shortening of the duration of immobility. DMPX at a dose of 3 mg/kg had no statistically significant influence on mice behavior in the FST (one-way ANOVA: *F*(3,36) = 13.48, *p < 0.05, **p < 0.01, *p* > 0.05, respectively).

#### Effect of Combined Administration of DMPX and Tested Antidepressants in TST

##### Effect of Combined Administration of DMPX and Imipramine in TST

DMPX (3 mg/kg) and imipramine (15 mg/kg), administered alone, did not affect immobility in the TST (*p* > 0.05) in mice. However, when given together, the duration of mouse immobility was significantly shortened (*p* < 0.001) (Fig. 2b).

Two-way ANOVA indicated a significant effect of imipramine (*F*(1,32) = 15.54, *p* = 0.0004), a significant effect of DMPX (*F*(1,32) = 36.92, *p* < 0.0001), and a significant interaction between imipramine and DMPX (*F*(1,32) = 9.736, *p* = 0.0038).

##### Effect of Combined Administration of DMPX and Escitalopram in TST

DMPX (3 mg/kg) and escitalopram (2 mg/kg), administered alone, did not affect immobility in the TST (*p* > 0.05) in mice. However, when given together, the duration of mouse immobility was significantly shortened (*p* < 0.001) (Fig. 2b).

Two-way ANOVA showed no effect of escitalopram (*F*(1,33) = 0.5905, *p* = 0.4477), a significant effect of DMPX (*F*(1,33) = 31.61, *p* < 0.0001), and a significant interaction between escitalopram and DMPX (*F*(1,33) = 6.251, *p* = 0.0176).

##### Effect of Combined Administration of DMPX and Reboxetine in TST

DMPX (3 mg/kg) and reboxetine (2.5 mg/kg), administered alone, did not affect immobility in the TST (*p* > 0.05) in mice. However, when given together, the duration of mouse immobility was significantly shortened (*p* < 0.001) (Fig. 2b).

Two-way ANOVA showed a significant effect of reboxetine (*F*(1,34) = 6.851, *p* = 0.0131), a significant effect of DMPX (*F*(1,34) = 21.82, *p* < 0.0001), and no interaction between reboxetine and DMPX (*F*(1,34) = 3.640, *p* = 0.0649).

### Spontaneous Locomotor Activity

#### Effect of DMPX on Locomotor Activity in Mice

The effect of DMPX (3, 6, and 12 mg/kg) on the spontaneous locomotor activity in mice is shown in Table 1. Statistical analysis of the results demonstrated that DMPX at doses of 3, 6, and 12 mg/kg had no statistically significant effects on animal’s locomotor activity versus the control group (one-way ANOVA: *F*(3,28) = 1.467, *p* > 0.05).

**Table 1:** Effect of DMPX on spontaneous locomotor activity in mice

Treatment (mg/kg)	Distance traveled (cm)
Saline (control group)	525.1 ± 79.04
DMPX 3	754.0 ± 72.81
DMPX 6	690.8 ± 62.92
DMPX 12	687.1 ± 102.5

DMPX and saline were administered i.p. 30 min before the test. Distance traveled was recorded between the second and the sixth min of the test. Each experimental group consisted of eight animals. The data are presented as the means ± SEM. The results were considered statistically significant if *p* < 0.05 (one-way ANOVA followed by Dunnett’s post hoc test)

#### Effect of Combined Administration of DMPX and Tested Drugs on Locomotor Activity in Mice

The effect of combined administration of DMPX and the antidepressants on locomotor activity is shown in Table 2. DMPX, imipramine, escitalopram, and reboxetine given alone or in combination had no statistically significant effects on mice locomotor activity.

**Table 2:** Effect of treatments on spontaneous locomotor activity in mice

Treatment (mg/kg)	Distance traveled (cm)
Saline + saline (control group)	640.7 ± 74.67
DMPX 3 + saline	955.8 ± 121.8
Imipramine 15 + saline	524.8 ± 96.23
DMPX 3 + imipramine 15	685.2 ± 102.9
Escitalopram 2 + saline	706.5 ± 110.8
DMPX 3 + escitalopram 2	1007 ± 145.2
Reboxetine 2.5 + saline	611.1 ± 163.9
DMPX 3 + reboxetine 2.5	755.2 ± 81.50

Antidepressants and saline were administered i.p. 60 min, whereas DMPX i.p. 30 min before the test. Distance traveled was recorded between the second and the sixth min of the test. Each experimental group consisted of eight animals. Data are presented as the means ± SEM (two-way ANOVA followed by Bonferroni’s post hoc test)

Two-way ANOVA demonstrated:

- (A): no effect of imipramine (*F*(1,26) = 3.396, *p* = 0.0768), a significant effect of DMPX (*F*(1,26) = 5.144, *p* = 0.0319), and no interaction (*F*(1,26) = 0.5452, *p* = 0.4669).
- (B): no effect of escitalopram (*F*(1,26) = 0.2310, *p* = 0.6348), a significant effect of DMPX (*F*(1,26) = 6.356, *p* = 0.0182), and no interaction (*F*(1,26) = 0.003379, *p* = 0.9541).
- (C): no effect of reboxetine (*F*(1,26) = 0.08845, *p* = 0.3556), no effect of DMPX (*F*(1,26) = 3.523, *p* = 0.0718), and no interaction (*F*(1,26) = 0.4887, *p* = 0.4907).

### Pharmacokinetic Studies

Pharmacokinetic studies outcomes are shown in Table 3. In the case of combined administration of DMPX with imipramine, no significant changes in drugs concentration were observed in murine serum and brain homogenates. DMPX increased the concentrations of escitalopram and reboxetine (*t* test: *p* < 0.05) in serum without significant changes in brain tissue.

**Table 3:** Effect of DMPX on the concentration of antidepressants in mouse serum and brain

Treatment (mg/kg)	Antidepressant concentration in serum (ng/ml)		Antidepressant concentration in brain (ng/g)	
Imipramine 15 + saline (metabolite-desipramine) Imipramine 15 + DMPX 3 (metabolite-desipramine)	229.5 ± 31.92 (39.20 ± 4.72) 215.0 ± 38.64 (40.66 ± 8.23)	*p* = 0.7767 *p* = 0.8826	6193 ± 466.5 (299.0 ± 42.22) 7166 ± 1523 (183.0 ± 39.19)	*p* = 0.5490 *p* = 0.3968
Escitalopram 2 + saline Escitalopram 2 + DMPX 3	54.87 ± 3.76 77.55 ± 6.72 *	*p* = 0.0109	639.1 ± 69.29 780.5 ± 103.9	*p* = 0.2721
Reboxetine 2.5 + saline Reboxetine 2.5 + DMPX 3	91.90 ± 7.60 126.3 ± 11.70 *	*p* = 0.0278	226.7 ± 30.82 159.0 ± 11.59	*p* = 0.0546

Antidepressants were administered i.p. 60 min, whereas DMPX i.p. 30 min before decapitation. Each experimental group consisted of 10 animals. Results are presented as mean values ± SEM. *p < 0.05 versus the respective control group (Student’s *t* test)

## Discussion

Adenosine system’s capability to regulate a large number of physiological processes including the CNS activity is well-documented (Jenner 2003; Jenner et al. 2009; Perez-Lloret and Merello 2014). The amount of adenosine determines whether adenosine suppresses or intensifies neurotransmission in the CNS. Under physiological conditions, adenosine levels in the brain are maintained at 25–250 nM. The A_1_Rs and A_2A_Rs are the main subtypes involved in the regulation of mental disorders, including anxiety or depression (Ruby et al. 2011). A_1_Rs and A_2A_Rs have a greater binding affinity for adenosine than the A_2B_R and A_3_R (10–100 nM, 1–5 mM, respectively) (Fredholm et al. 1999, 2005a, b; Fredholm 2010; Ruby et al. 2011). The highest density of A_1_Rs is found in neurons of cortex, hippocampus, cerebellum, as well as in dorsal horn of spinal cord (Mahan et al. 1991; Dixon et al. 1996; Fredholm et al. 2000). These receptors participate in the tonic inhibition of neuronal activity. Presynaptic A_1_Rs regulate the release of neurotransmitters, whereas postsynaptic A_1_Rs modulate the activity of K^+^ channels (Ebersolt et al. 1983; Linden 1991; Linden et al. 1991; Heurteaux et al. 1995). Adenosine A_2A_Rs are expressed mainly in the dorsal and ventral striatum and olfactory tubercle (Schiffmann et al. 1991a, b; Fink et al. 1992; Svenningsson et al. 1997a, b; Rosin et al. 1998). They exert a stimulating effect on neurons, increasing the level of cAMP in the CNS. It is also known that A_2A_Rs bind to other neurotransmitter receptors, including dopamine (mainly D_2_) and glutamate receptors. Such receptor-receptor interaction seems to be necessary for striatal function and may be impaired in mental diseases (Ferré et al. 1992, 1996a, b, 1997; Ferré and Fuxe 1992). A growing evidence supports their influence on behavior, mood, and cognitive function, which results from a close relationship between the adenosine modulation and the dopaminergic and glutamatergic transduction (Fredholm et al. 2005a).

It was found that both adenosine and its analogs (e.g., 2-chloroadenosine) increased the immobility time in the FST, which equates to behavioral despair (Duman 2010). On the contrary, the non-selective inhibition of ARs in the CNS seems to induce antidepressant-like behavior in animals. One of the tested non-selective AR antagonist was caffeine, which in a dose-dependent manner effectively prolonged a mobility period in the FST (El Yacoubi et al. 2003; Szopa et al. 2016). Research aimed at determining whether selective genetic or pharmacological inhibition of A_2A_Rs will contribute to the antidepressant-like effect has been carried out recently (El Yacoubi et al. 2001, 2003; Yamada et al. 2013). The suppression of the behavioral despair was observed by El Yacoubi et al. (2001) in mice with the genetically inactive A_2A_Rs. In turn, Coelho et al. (2014) demonstrated that the rodents overexpressing A_2A_Rs in the CNS structures, such as cortex, striatum, and hippocampus, exhibit symptoms of depression. The period of immobility in the FST and TST was shortened, inter alia, by the acute oral administration of istradefylline (Yamada et al. 2013) and i.p. injection of single dose of ZM241385 or SCH58261 (El Yacoubi et al. 2001, 2003). Here, the dose-dependent antidepressant-like effect, in both the FST and TST, has been demonstrated for another A_2A_R antagonist, i.e., DMPX, which displays higher selectivity for A_2A_ receptors over A_1_ receptors. Of the three tested doses (3, 6, and 12 mg/kg), only the lowest dose did not change the mice behavior. Also, subchronic and chronic administration of a selective antagonist of A_2A_R, istradefylline, is characterized by a dose-dependent effect in the FST (studied dose range 0.16–2.5 mg/kg, per os) (Yamada et al. 2013) and the learned helplessness test (LH) in the rat (studied dose range 0.31–5.0 mg/kg, per os) (Yamada et al. 2014).

However, there is lack of information about the interaction between A_2A_R antagonists and therapeutic agents commonly used in the treatment of patients with depression. Monoamine hypothesis of depression assumes that the essential reason of depressive symptoms is a diminution of NA, 5-HT, and DA level in the CNS (Akiskal and McKinney 1973; Delgado 2000). Therapeutic agents that elevate the levels of these monoamine transmitters have been shown to be effective in the depressive disorders (Gillman 2007). The stimulation of the monoaminergic system, notably noradrenergic and serotonergic, by A_2A_R blockade may also produce a similar antidepressant effect (Yamada et al. 2014). In our studies, we have demonstrated the synergism of the antidepressant-like activity of the A_2A_R antagonist, DMPX, and selected antidepressants from various therapeutic groups. DMPX, imipramine, escitalopram, and reboxetine were injected at doses that did not affect the animals’ behavior in the FST, TST, and locomotor activity test. Concomitant administration of DMPX with these agents resulted in a significant stimulation of mice motility either in the FST or TST. The reliability of the obtained data is supported by the fact that observed effects did not correspond with increase in the mice spontaneous locomotor activity. This means that observed antidepressant-like activity of the studied agents is not a false positive.

It has been previously demonstrated that non-selective AR antagonist, caffeine, at a non-effective dose (5 mg/kg) potentiated the activity of antidepressants belonging to different classes (i.e., fluoxetine, paroxetine, escitalopram, imipramine, desipramine, reboxetine, venlafaxine, moclobemide, mianserin, milnacipran, bupropion, and agomelatine) (Kale and Addepalli 2014; Poleszak et al. 2015, 2016b; Szopa et al. 2016, 2017). The antidepressant-like effect of the analyzed drug-drug combinations (DMPX-imipramine, DMPX-escitalopram, and DMPX-reboxetine) observed in our study is probably the result of the sum of their action on the monoaminergic transmission. Moreover, it is well-known that stress-induced illnesses, such as depression, are closely related to the hypothalamic-pituitary-adrenal axis (HPA) (Pariante and Miller 2001; Pariante and Lightman 2008). For example, maternal separation, which is a model of depression (Vetulani 2013), was shown to increase corticosterone plasma levels (Biagini et al. 1998). The modification of this hormonal system activity plays an essential role in the action of TCAs and SSRIs (Reul et al. 1993, 1994). The release of steroid stress hormone, e.g., cortisol, corticosterone, is controlled by the adenosinergic system (Scaccianoce et al. 1989; Chau et al. 1999). Yamada et al. (2013) showed that injection of corticosterone suppressed the antidepressant-like behavior observed in rats after administration of A_2A_R antagonists. Accordingly, A_2A_R antagonists through direct and/or indirect modulation of the HPA axis can influence stress-induced diseases (Yamada et al. 2014). Therefore, the effects on the HPA axis may be another underpinning of the behavioral effects following concomitant administration of DMPX and antidepressants. Because antidepressants were shown to produce different effects on the HPA axis (depending on the primary mechanism of action/acute or chronic administration), it may be hypothesized that adding adenosine-mediated impact on the stress axis to the effects of antidepressant drugs on this axis, may be beneficial in terms of depression treatment.

Because DMPX is a caffeine analog, it is probable that its pharmacokinetics is similar to that of other xanthines. DMPX exhibits 100% bioavailability after both i.p. and per os administration (Yang et al. 2007). The main xanthine-metabolizing isoenzyme is CYP1A2. This isoenzyme is also engaged in the biotransformation of most drugs, including psychotropics (Caccia 1998; Nelson et al. 2004; Guengerich 2008; Zanger et al. 2008). Therefore, there was a high expectation of an interaction between DMPX and antidepressants: imipramine, escitalopram, and reboxetine. There is no information on the metabolism of DMPX either in rodents or in humans. Based on the data collected during the HPLC analysis, we found that DMPX does not significantly affect the level of imipramine, as well as desipramine (an active metabolite of imipramine) in mice. Similarly, in our previous studies, in which caffeine was used in the same scheme, there were no changes in serum concentration of imipramine (Szopa et al. 2016). The combined use of caffeine and reboxetine or escitalopram also did not contribute to the altered levels of these antidepressants in serum of mice (Szopa et al. 2016). Notwithstanding, in the case of a concomitant administration of DMPX with reboxetine and escitalopram, a small, but statistically significant enhancement in their concentrations in serum was found. Escitalopram and reboxetine levels in brain tissue did not increase/decrease statistically when administered with DMPX. It is astounding that the changes in levels of escitalopram/reboxetine in murine serum did not reflect the changes in their levels in brain homogenates. The reason for this may be a delay in the transport of these drugs across the blood-brain barrier (Burke and Preskorn 2004). The source of DMPX-escitalopram and DMPX-reboxetine interactions shown in our study is not entirely clear. The results imply that the interaction between DMPX and imipramine probably takes place in the pharmacodynamic phase, i.e., at the neurotransmitter or receptor level, etc.

## Conclusions

Based on our and quoted in this paper outcomes, it can be inferred that the adenosine system plays a role in several animal tests for depression and the selective inhibitors of A_2A_R reverse this behavior. The present study supports the possibility of augmenting the antidepressant pharmacotherapy with DMPX or different A_2A_R antagonists. The interaction between the tested xanthine analog and imipramine seems to be exclusively pharmacodynamic in nature, whereas an increased antidepressant activity of escitalopram/reboxetine was at least partly related to its pharmacokinetic interaction with DMPX. In summary, the presented results indicate that A_2A_R may be a useful target for the therapy of depressive disorders. A_2A_R antagonist can be an advantageous complement to the current antidepressant pharmacotherapy, but it is necessary to perform further studies, which shall explore this issue.
